# Rapidly Establishing an Ultra-Cold Supply Chain of Vaccines in Israel: Evidence for the Efficacy of Inoculation to Mitigate the COVID-19 Pandemic

**DOI:** 10.3390/vaccines11020349

**Published:** 2023-02-03

**Authors:** Michael Naor, Gavriel David Pinto, Pini Davidov, Lina Abdrbo

**Affiliations:** 1 School of Business Administration, Hebrew University, Jerusalem 9190501, Israel; 2 Industrial Engineering and Management, Azrieli College of Engineering, Jerusalem 9103501, Israel; 3 UNEC Cognitive Economics Center, Azerbaijan State University of Economics, Baku AZ1001, Azerbaijan

**Keywords:** COVID-19, ultra-cold supply chain, Israel, vaccine efficacy

## Abstract

The agenda of this research was to investigate how to mitigate the spread of coronaviruses by rapidly establishing an ultra-cold supply chain of vaccines. Data analysis was conducted by linear regression utilizing a dataset publicly available from the Israel Ministry of Health regarding the daily rates of people vaccinated, tested, hospitalized, etc., since the start of the pandemic. The data provide statistical evidence for the efficacy of the Pfizer vaccines in diminishing a wide variety of disease factors, such as the number of patients who were lightly, moderately, or severely sick, and daily deaths, as well as the rate of spread (R-ratio) and number/percentage of people infected. Insightfully, the data corroborate how the first and second doses of the vaccines were able to decrease the wave of COVID-19, which hit Israel in January 2021, while the booster third dose was able to diminish a subsequent COVID-19 wave occurring in Israel in July 2021.

## 1. Introduction

The pandemic transformed old-fashioned pharmaceutical supply chain networks because of the need to overcome the traditional hurdles of red tape to enable a fast response, which was essential as the public was under state of panic [1]. According to the World Health Organization [2], an ultra-cold chain (UCC) is needed for COVID-19 vaccines because of their requirement to be stored at ultra-low temperatures (ULT) of between −80 °C and −60 °C. While the pharmaceutical industry has an abundance of experience with the pipelines of common vaccines, the distribution of an ultra-cold supply chain introduces an array of new challenges, such as the need for specialized packaging, shipment, and warehouses coupled with enhanced tracking for the handling of materials and border administration between countries. Because low-to-middle income countries do not have UCC capability, on behalf of COVID−19 Vaccines Global Access (COVAX), UNICEF acquired freezers which can store up to 300,000 doses each.

Israel rolled out its vaccination project for the emerging pandemic on 20 December 2020. The Israeli government attempted to secure contracts to procure the vaccine from global pharmaceutical companies because an Israeli-developed vaccine, called BriLife, was, in 2020, in the first clinical trial stage [3,4]. By the time the United States Food and Drug Administration (FDA) issued an emergency use approval for the Pfizer-BioNTech COVID-19 vaccine, Israel had reached an agreement with Pfizer to purchase and receive millions of doses that would cover its full population. After fruitful negotiations between the Israel Prime Minister, Binyamin Netanyahu, and Pfizer’s chief executive officer, Albert Bourla, in the year 2020, a contract was signed. The ulterior motive behind Pfizer’s decision to sign the contract stemmed from the fact that under Israeli law, all residents are insured with one of the four publicly funded health organizations (Clalit, Leumit, Maccabi, and Meuhedet), all of which are managed by a centralized system containing the digital medical records of the entire country’s population. The government consent for accessibility to such data by Pfizer may have been fruitful for generating valuable information assessing the clinical efficacy of the COVID-19 vaccines, for example, with respect to their effects on patients with certain medical conditions or on different demographic groups. In the United States of America, a majority of the population is insured by various private vendors that do not pool medical information in a centralized database, making such analysis cumbersome [5]. Since 2006, federal laws have required the Department of Health and Human Services to develop a real-time electronic network that would improve the nation’s situational awareness during a public health emergency [6]. This network was meant to be used to facilitate the early detection of, and rapid response to, potentially catastrophic events. However, 15 years later, this network is still not operational [7].

**Figure 1:**
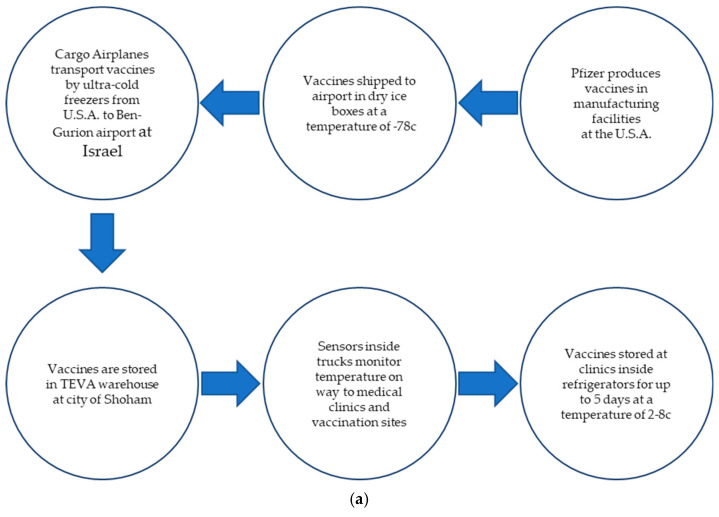

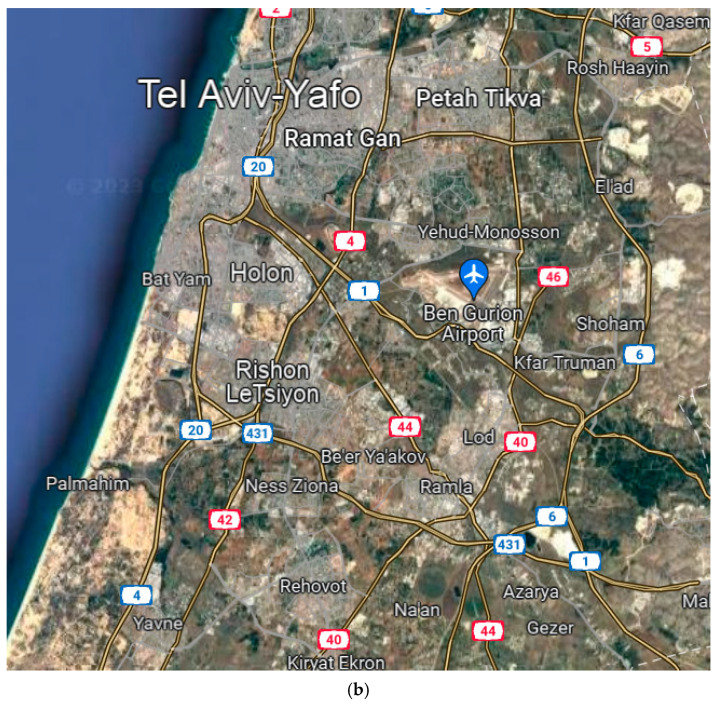
(**a**) The ultra-cold supply chain for the COVID-19 vaccines (**b**) Map of Israel showing Road 6, the city of Shoham, and the Ben-Gurion airport.

The entire operation of shipping the vaccines overseas from the United States to Israel (Figure 1a) was managed and supervised by a branch of the Israel Defense Forces titled the Israel Home Front Command (in Hebrew, Pikud Oref). This helped to streamline the aviation process and distribute the vaccines between the four Israeli healthcare providers [8]. The Home Front Command played a vital role in establishing testing centers, enforcing lockdown restrictions, and administering vaccines in key areas such as the orthodox city of Bnei Brak, which experienced high rates of infection in Israel because of its large average household large size and its crowded learning environments in Jewish learning facilities (Yeshiva). Contact tracing was conducted by a center titled Alon, which was run by Israeli soldiers. It should be mentioned that during years 2020 and 2021, the eligible population for vaccination comprised adults, with preference given to people sixty years of age or older, nursing home residents, and others at high risk of severe medical conditions, as well as frontline medical employees. The permission to start vaccinating of young adults was bestowed much later, in 2022, and there is an ongoing discussion within the Ministry of Health regarding the necessity of vaccinating toddlers.

The Pfizer vaccines were shipped to Israel via air transport in specially designed, temperature-controlled containers utilizing dry ice to maintain the recommended storage temperature conditions of approximately −70 °C. The thermally monitored containers were then transported by truck from the airport to a state-of-the-art technology logistics warehouse (Marlog, in Hebrew) operated by the SLE Ltd. (named after the founders, Salomon, Levin, and Elstein) pharmaceutical supply chain conglomerate in the city of Shoham, where the vaccines were stored in freezers.

The SLE logistics center is a 50,000-square-meter sustainable building founded in 2011. Its dimensions are over 400 m in length, with a height of 42 m, with 84 unloading docks. Storage and assembly within the facility are carried out using 11 cranes and conveyors with total length of 2.1 km. It can sort approximately 1 million orders (20,000 catalog numbers), deliver 100 million items annually to the Israeli market (1000 packages per hour), and is capable of storing approximately 75,000 pallets in various configurations and temperatures. To ensure business continuity and safety, each product is divided between two storage areas that mirror each other in terms of content, which are set 150 m apart and have a dual power supply and water feed (an emergency generator provides an alternate source of power 24 h per day, 7 days per week). An information center backs up the servers’ data in a secured basement located two levels underground [9].

The city of Shoham (see map in Figure 1b) was chosen for three reasons that make it a strategic location: it is positioned (1) at the center of Israel, (2) near the Ben-Gurion Israel major international airport, and (3) in proximity to the major Israel highway (Road 6) for rapid access by the trucks.

From the SLE warehouse at Shoham, the vaccines were packaged and transported under strict temperature monitoring to hospitals, clinics, and designated immunization centers around the country. Each truck’s temperature was monitored by sensors from a control center to ensure the vaccines were under supervision, as Israel has advanced Internet-of-Things technology as a startup nation [10]. To illustrate the rapid distribution process, by the end of 2020, the State of Israel, with a population of 9.3 million, delivered more COVID-19 vaccine doses than all other countries aside from China, the US, and the UK. Moreover, Israel had administered almost 11.0 doses per 100-person populations, while the next highest rates were 3.5 (in Bahrain) and 1.4 (in the United Kingdom). All other countries had administered less than 1 dose per 100-person populations [11].

Since 1995, Israel’s National Health Insurance (NHI) law has ensured public coverage for all citizens and permanent residents. Under the Israeli National Health Insurance Law, membership in one of the four following health providers is mandatory for all residents of Israel: Clalit, Maccabi, Meuhedet, and Leumit. Clalit is the biggest of the four agencies, with approximately 50% of the population having membership. The COVID-19 vaccination process was administered through these four health providers primarily by certified trained nurses and, in some cases, by workers affiliated with Magen David Adom (Israel’s national emergency medical, disaster, ambulance, and blood bank service). Thawed glass vials of vaccines could be stored at the clinics and vaccination sites inside medical refrigerators for up to 5 days at a temperature of 2–8 °C.

Appointments for vaccinations were scheduled digitally through the four health providers’ websites. Because Israel’s size is small, the health agencies were able to provide coverage for the entire country’s population.

The purpose of the current case study utilizing a reliable dataset that is publicly available in the State of Israel by the Ministry of Health is to examine how cooperation with the vaccine manufacturer, Pfizer, created an ultra-cold supply chain of vaccines that caused a decrease in the spread of the disease such that the economy could be reopened without closure and people return to their workplace employment location headquarters [12]. Specifically, the study’s goal was to investigate two research questions: (1) how can a country rapidly establish an ultra-cold supply chain for vaccines? (2) What was the impact of the coronavirus vaccine doses on Israel’s morbidity indices?

The statistical analysis examined by regressions the impact of the gradual vaccination process with a series of jabs, namely, dose 1, dose 2, and dose 3 (booster), on the amount of patients with severe, moderate, and mild levels of COVID-19. Lessons gleaned from the study can help to overcome resistance among people for vaccination by substantiating its significant effectiveness to mitigate the pandemic. Further, delineating Israel’s rapid response serves as framework to guide developing countries on how to design an ultra-cold supply chain of vaccines as new strains of COVID-19 emerge.

## 2. Materials and Methods

### 2.1. Interviews

Table 1 describes information about the supply chain personnel that were interviewed during the research because they were involved in the distribution and logistics of the vaccines in Israel.

Table 2 describes the questionnaire items used to interview the supply chain managers. Similarly, Table 3 delineates the questionnaire items used to interview the personnel in healthcare clinics.

To gain firsthand experience for the verification of the information collected during interviews, the authors were hosted by Teva to perform a plant tour.

### 2.2. Applied Statistical Methods

The dataset is curated from a highly reliable COVID-19 data tracker published by the Israeli government [13] which has been utilized by numerous scholars to publish research in top-tier journals [14]. The data tracker tracks daily the number of people that contract COVID-19 and are diagnosed at various levels of sickness (light, medium, and severe). It also records the spread of virus transmission among the population and the number of deaths. The analysis was conducted using the business analytics software, R, version 4.0.3. [15].

## 3. Results

**Figure 2:**
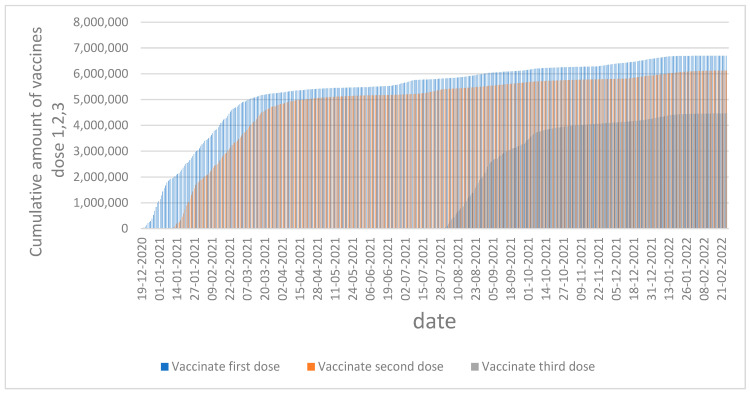
Cumulative amounts of vaccine doses 1, 2, and 3 by date in Israel.

Figure 2 illustrates the number of people vaccinated by doses 1, 2, and 3 over time. From the graph, an insight emerges that while the first and second doses were in high demand, with over six million does, the third dose was less acceptable among the population, with less than five million inoculations.

**Figure 3:**
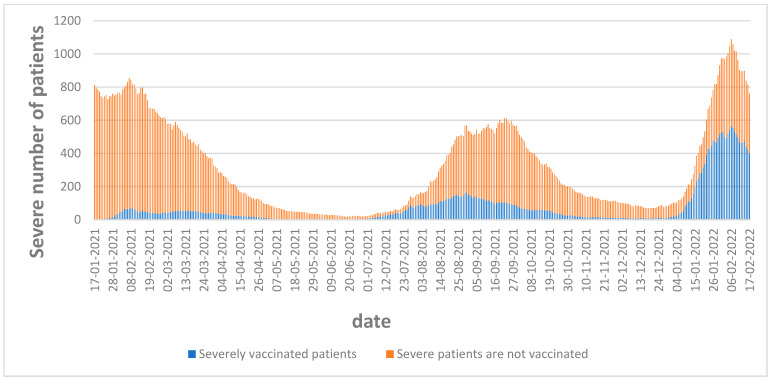
Severely ill vaccinated versus unvaccinated people.

Figure 3 is of major importance in this study because it visually highlights that most people who were severely sick were non-vaccinated. In the next section, this finding is statistically substantiated using regression analysis.

**Figure 4:**
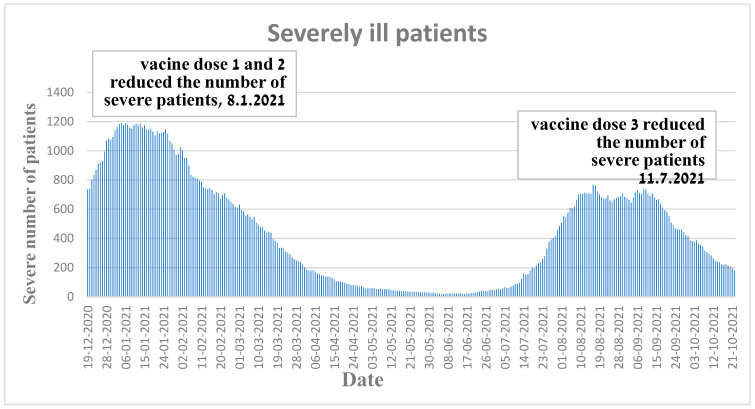
Amount of severely ill patients in Israel during two waves.

Figure 4 illustrates in a visual graphical fashion how the administration of the first vaccine dose combined with second dose of Pfizer mitigated a COVID-19 wave which started on 8 January 2021. These results are consistent with the findings of Haas et al. [16].

Insightfully, Figure 4 also corroborates how the third dose (booster) of the COVID-19 vaccine was able to diminish a wave of COVID-19 (the delta variant) which started in Israel on 11 July 2021. These results are consistent with the findings of Muhsen et al. [17] from nursing homes.

**Figure 5:**
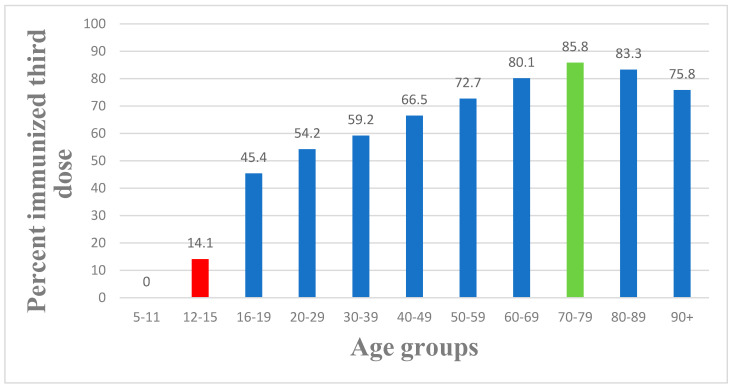
Percentage of people by age who had received the Pfizer booster dose.

Figure 5 illustrates the percentage of people by age who had received the Pfizer booster dose. Based on the graph, more than 80% of people in the age group 60–80 were vaccinated, which explains the ability of the booster to have reduced the amount of severely ill patients during the wave which began on 11 July 2021.

**Figure 6:**
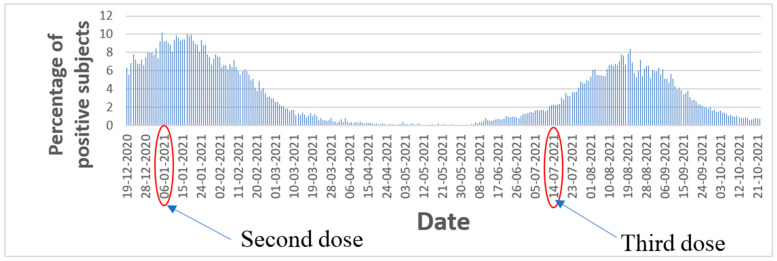
Percentages of positive tests.

**Figure 7:**
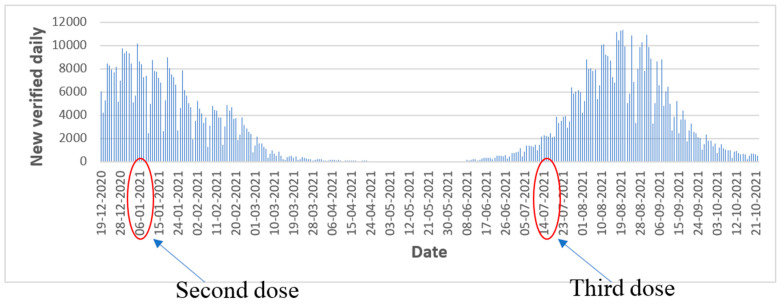
New daily cases.

Figure 6 illustrates the percentage of people who tested positively. Based on the graph, the number of people who tested positively was reduced following administration of the first and second doses during the wave in the beginning of the year 2021. Similarly, the booster was able to diminish the number of people who tested positively during the wave towards end of year 2021. The ability of the booster to reduce the amount of severely ill patients during the wave that began on 11 July 2021 is illustrated. The same pattern is evident in Figure 7, which shows the amount of new daily cases.

Table 4 provides information about the distribution of vaccinations by city. Key insights emerge from the table. For example, in cities with a high Arab population (i.e., Nazareth), the percentage of vaccinations was lower. In addition, the percentage of people vaccinated in cities with strong orthodox communities, such as Jerusalem, is substantially lower compared to secular cities, such as Tel Aviv. Finally, the percentage of people who received the third dose (booster) is substantially lower than those of the people who received the first and second doses in all cities.

**Figure 8:**
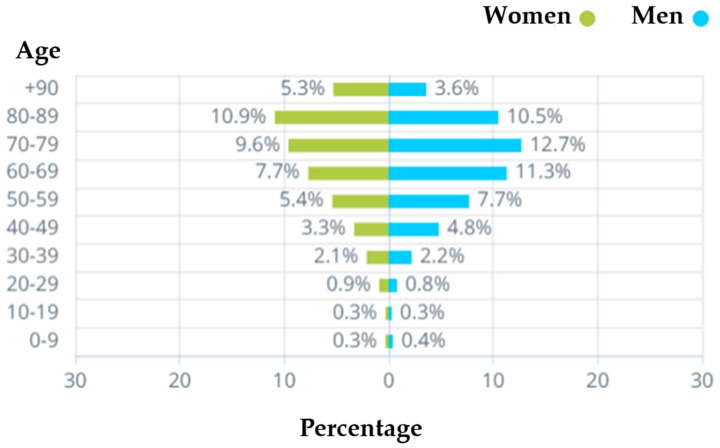
Demographics of severely ill people by age and gender (up to 13 June 2022).

Figure 8 juxtaposes the demographics of the severely ill patients by age and gender. An important insight that emerges is that the percentage of severely ill men is significantly higher (more than 30%) compared to severely ill women across the following age clusters: 40–49 (women 3.3% vs. men 4.8%), 50–59 (women 5.4% vs. men 7.7%), 60–69 (women 7.7% vs. men 11.3%), and 70–79 (women 9.6% vs. men 12.7%). This result is consistent with numerous studies worldwide which have found that men are at higher risk of becoming severely sick and developing life-threatening complications compared to women [18]. In terms of age, younger age clusters such as 20–29 and 30–39 were significantly less likely to become severely ill compared with people above age of 40, which is consistent with previous worldwide studies [19]. Specifically, according to Figure 8, the cumulative percentage of severely ill people in the age cluster 0–39 is 7.3%, whereas the aggregate percentage of severely ill people above age 40 is 92.7%.

There is a need to construct three correlation tables because dose 2 was administered one month after dose 1, and dose 3 was administered five months after dose 1 and 2, making it unfeasible to run a correlation for all variables simultaneously. Table 5 validates a negative significant correlation between dose 1 of the vaccine with all the sickness factors (mild, moderate, and severe sickness), as well as with daily deaths, infection rates (R), and the percentage and number of people who tested positive). Similarly, Table 6 corroborates a negative significant correlation between dose 2 of the vaccine with all the sickness factors (mild, moderate, and severe sickness), as well as with daily deaths, infection rates (R), and the percentage and number of people who tested positive). The booster dose 3 in Table 7 verifies the previous findings, showing a negative significant correlation with all the sickness factors (mild, moderate, and severe sickness), as well as with daily deaths, infection rates (R), and the percentage and number of people who tested positive).

Next, a least squares regression analysis was conducted using the statistical software R, version 4.0.3. The dependent variables included morbidity indices such as the number of severely, moderately, and mildly sick patients diagnosed or hospitalized. The independent variables included the cumulative number of people vaccinated by the first and second doses and the third dose (the booster).

Because of multicollinearity between the independent variables (for example, the correlation between the aggregate number of people inoculated by dose 1 and the aggregate number of people inoculated by dose 2 was 0.99), the regression analysis was conducted in a stepwise process for each variable individually. In terms of the units of analysis, because the number of people hospitalized was counted in hundreds and the cumulative number of people vaccinated was counted in millions, the regression coefficients appearing inside the tables were multiplied by 100,000 to normalize the results. On the one hand, the infection rate (R) was assessed as a single digit number which was regressed against the number of vaccinated people in millions, which explains the reason why the regression coefficient was a small number after being multiplied by 100,000 (compared to the regression coefficients of the number of hospitalized people, which was counted in hundreds or thousands). On the other hand, the daily number of people infected was counted in thousands, which again explains the large regression coefficient that resulted when it was regressed against the number of people inoculated by doses 1, 2, and 3.

Table 8 indicates that the aggregate number of people inoculated by dose 1 explains 89.4% of the variance in the severely sick patients. Dose 2 accounts for 96% of the variance in the severely sick people. Dose 3 accounts for 43% of the variance in the severely sick people.

Table 9 indicates that the aggregate number of people inoculated by dose 1 explains 90% of the variance in the moderately sick patients. Dose 2 accounts for 93.7% of the variance in the moderately sick people. Dose 3 accounts for 64.5% of the variance in the moderately sick people.

Table 10 indicates that the aggregate number of people inoculated by dose 1 explains 91.8% of the variance in the mildly sick patients. Dose 2 accounts for 95.9% of the variance in the mildly sick people. Dose 3 accounts for 66% of the variance in the mildly sick people.

Table 11 indicates that the aggregate number of people inoculated by dose 1 explains 90.9% of the variance in the daily deaths. Dose 2 accounts for 90.5% of the variance in the daily deaths. Dose 3 accounts for 58.5% of the variance in the daily deaths.

Table 12 indicates that the aggregate number of people inoculated by dose 1 explains 24.4% of the variance in the daily infection rate (R). Dose 2 accounts for 10.2% of the variance in the daily infection rate. Dose 3 accounts for 67.3% of the variance in the daily infection rate. It is interesting to note that the values in Table 6 for the daily infection rates are substantially lower than the values for the sickness factors described in the other regression tables, which sheds light on the reasons for the Israeli Ministry of Health government officials’ and the Czar of Corona’s decisions pertaining to the lockdowns and mask mandates, which were made based on the empirical data on the hospitalization rates of severely ill people or deaths, rather than on the daily infection rates.

Table 13 indicates that the aggregate number of people inoculated by dose 1 explains 93.4% of the variance in the daily percentage of people who tested positive. Dose 2 accounts for 98% of the variance in the daily percentage of people who tested positive. Dose 3 accounts for 71% of the variance in the daily percentage of people who tested positive.

Table 14 indicates that the aggregate number of people who were inoculated by dose 1 explains 85.6% of the variance in the daily number of people who tested positive. Dose 2 accounts for 86.1% of the variance in the daily number of people who tested positive. Dose 3 accounts for 59.2% of the variance in the daily number of people who tested positive.

## 4. Discussion

The necessity for verifying the efficacy of the Pfizer COVID-19 vaccines stemmed from the doubts cast by the anti-vaccine movement [20]. The merits of the current research are threefold.

First, it will help to encourage people to become vaccinated, despite their hesitance to do so [21], by statistically validating the vaccine’s efficacy in mitigating the pandemic wave in the beginning of 2021 across an array of sickness factor levels. The results show a negative relationship between inoculation doses 1 and 2 and all sickness factors on country-wide scale using a reliable dataset officially published by the Israel Health Ministry, a result which is consistent with previous study findings (Rossman et al., 2021).

Second, the results validate the necessity for administering a third dose (a booster) of the vaccine to mitigate the new variant (Delta), which emerged towards the end of 2021, because the breakthrough cases of COVID-19 (where vaccinated people were reinfected) occurred as the effectiveness of dose 2 was waning after a time period of approximately five months [22,23]. The emergence of new, highly infectious variants of COVID-19, such as delta and omicron, implies that the herd immunity threshold is a moving target which is difficult to meet, even after over 60% of Israel’s population had achieved vaccination status [24].

Third, the successful Israeli paradigm of emergency response can be used as a template for countries interested in following in Israel’s footprints for creating a rapid process to establish an ultra-cold supply chain for vaccines before new variants emerge [25]. While the ability to access patients’ records that are registered at various healthcare providers which do not pool medical information has been a bottleneck in the effort to coordinate a testing and vaccination campaign worldwide [26], Israel’s centralized healthcare database for the entire population has proven to be efficient at tracking healthcare data about the pandemic spread and the pace of vaccination. This may require governmental regulatory action to institutionalize laws allowing for the future sharing of information in countries where there is a large spectrum of public and private medical insurance providers.

An additional lesson gained is that there is a need to diminish the reliance on assessing herd immunity as the sole proxy for pandemic spread because its mathematical formula for calculation is based on the infection rate, which encompasses uncertainty in the case of COVID-19 as a consequence of testing inaccuracies, among other reasons [27]. To elaborate, infection rates are not measured directly; they are based on a mathematical model which assesses how many new people each COVID-positive person is going to infect, and, as such, infection rates represent estimations of a trend [28]. In Israel, for extended periods of time during the pandemic, the value of the infection rate was above 1.0, but the number of people hospitalized was stable. Consequently, governments worldwide should adopt a differential policy for easing lockdowns based on actual verified data such as recorded vaccination rates in various cities and hospitalization logs [29]. Israel was early in implementing this policy using a vaccination green pass certificate, which was required for entry into public facilities, and a traffic light model which ranked residential districts on a color scale of red, orange, yellow, and green levels of risk, along with associated restrictions [30]. At the peak of pandemic, and for a limited time under Israeli court scrutiny, digital contact tracing (the surveillance of transmission) was monitored using cell phone location data [31].

An interesting initiative undertaken by the Israeli government was the appointment of a Joint Commission of the Israel National Bioethics Council, the Ethics Bureau of the Israel Medical Association, and the Israeli Ministry of Health for the creation of a protocol for triaging severely ill patients in case the hospital network availability of beds was over capacity [32]. To gain public trust, the commission consisted of subject matter experts in ethical medicine assembled to develop triage criteria based on religious and gender diversity, transparency, and societal humanity values such as compassion. An alternative approach adopted, given Israel’s experience in deploying military field hospitals [33], was the establishment of the Military COVID-19 Wards (MCWs) operated by the Israeli Defense Force Medical Corps within the civilian Rambam hospital in the city of Haifa [34].

## 5. Conclusions

On 1 March 2022, the Israeli government put an end to the over-two-years-long COVID state of emergency, which was institutionalized at the start of pandemic, and after a three-month transition period, on 1 June all restrictions were officially lifted. Similarly, in the United States of America, on 18 September 2022, President Joe Biden declared that the COVID pandemic was over. On the global stage, the Director-General of the World Health Organization (WHO), Tedros Adhanom Ghebreyesus, postulated on 16 September 2022 that the end of pandemic was in sight because the number of weekly COVID deaths worldwide had reached its lowest point since March 2020, when the coronavirus was first declared a global pandemic [35].

## Figures and Tables

**Table 1:** List of interviews.

Company	Title of Subject Matter Expert Interviewed	Date
Eli Lilly and company	Expert in cold supply chain	2 February 2022
Teva conglomerate	Procurement manager	21 February 2022
Kupat Cholim Lehumit	CEO of logistics	3 March 2022
Kupat Cholim Clalit	Senior nurse in charge of vaccination process	21 February 2022

**Table 2:** Questionnaire for the interviews with the supply chain managers.

1.	What is your name and role in the company?
2.	How are you involved in the treatment of corona?
3.	Please explain how the vaccines were transported to Israel onboard the airplanes during flight?
4.	How long did it take to transport the vaccines from the Pfizer production plant to Teva’s warehouse?
5.	How many vaccines were packaged in each aircraft?
6.	At what temperature were the vaccines stored onboard the airplanes during flight?
7.	Where vaccines were stored in Israel, and in which city?
8.	Why did Teva choose the location of the logistics center in exactly this geographical place?
9.	What type of refrigerators were inside the logistics center and how many vaccines were stored in each refrigerator?
10.	Did employees wear a body heating suit because of the ultra-cold temperatures inside the warehouse?
11.	How long could a vaccine be stored in the refrigerator of the logistics center?
12.	How were the vaccines delivered to the health clinic, and how many were in each truck?
13.	How long could a vaccine remain in the health clinic before it expired?
14.	Was a vaccine that was almost expired thrown away or was it transferred to another country?
15.	Was vaccine waste recycled?
16.	What was unique about the ultra-cold supply chain?

**Table 3:** Questionnaire for the interviews with the employees at the health clinics.

1.	What was your position in the health clinic?
2.	What can you tell us about the vaccination process at the health clinic?
3.	How were patients summoned for vaccination?
4.	Is there a list of people who have been vaccinated on the HMO’s computer and how did people receive a message to come and get vaccinated—by phone or text message?
5.	Is there a central information system for all patients in Israel?
6.	Are the four health providers in Israel (Maccabi, Leumit, Meuhedet, and Clalit) sharing information about patients?
7.	Where were people vaccinated (stations, sport arenas, etc.)?
8.	How many people were vaccinated each day, on average?
9.	How many vaccines were in each bottle?
10.	How many bottles were in each tray stored in the refrigerators?
11.	At what temperature were the vaccines stored at in the health clinics?
12.	At what frequency each week were the vaccines shipped to the health clinics?
13.	How did the bottles arrive (whether they were in a box or a cooler) and how many were in each package?
14.	From which logistics center in the country were the vaccines shipped?
15.	Can you recommend additional people to talk with about vaccination?
16.	What was your position in the health clinic?

**Table 4:** Immunization by city in Israel (up to 13 June 2022).

City	% Third-Dose Vaccinated	% Second-Dose Vaccinated	% First-Dose Vaccinated
**Jerusalem**	34.22%	53.09%	61.7%
**Tel Aviv**	59.33%	73.42%	76.88%
**Haifa**	60.72%	75.07%	79.02%
**Beer Sheva**	55.79%	72.38%	77.26%
**Acre**	51.21%	70.53%	76.6%
**Shoham**	75%	89.97%	90%
**Eilat**	59.65%	79.26%	83.52%
**Nazareth**	36.03%	59.15%	68.28%

**Table 5:** Correlation between dose 1 and the sickness factors from 1 January 2021 to 21 May 2021.

	Dose 1 Aggregate Inoculation	Mildly Sick People	Moderately Sick People	Severely Sick People	Daily Deaths	Infection Rate (R)	Percentage of Positive Tests	Number of Positive Tests
Dose 1 Aggregate Inoculation	1	−0.958 **	−0.949 **	−0.946 **	−0.954 **	−0.494 **	−0.966 **	−0.925 **
Mildly Sick	−0.958 **	1	0.991 **	0.985 **	0.935 **	0.362 **	0.968 **	0.914 **
Moderately Sick	−0.949 **	0.991 **	1	0.983 **	0.932 **	0.339 **	0.966 **	0.920 **
Severely Sick	−0.946 **	0.985 **	0.983 **	1	0.933 **	0.328 **	0.971 **	0.897 **
Infection Rate (R)	−0.494 **	0.362 **	0.339 **	0.328 **	0.420 **	1	0.473 **	0.419 **
Percentage of Positive Tests	−0.966 **	0.968 **	0.966 **	0.971 **	0.937 **	0.473 **	1	0.920 **
Number of Positive Tests	−0.925 **	0.914 **	0.920 **	0.897 **	0.885 **	0.419 **	0.920 **	1

** *p* < 0.01.

**Table 6:** Correlation between dose 2 and the sickness factors from 13 January 2021 to 21 May 2021.

	Dose 2 Aggregate Inoculation	Mildly Sick People	Moderately Sick People	Severely Sick People	Daily Deaths	Infection Rate (R)	Percentage of Positive Tests	Number of Positive Tests
Dose 2 Aggregate Inoculation	1	−0.979 **	−0.968 **	−0.980 **	−0.951 **	−0.319 **	−0.990 **	−0.928 **
Mildly Sick	−0.979 **	1	0.988 **	0.988 **	0.922 **	0.234 **	0.967 **	0.909 **
Moderately Sick	−0.968 **	0.988 **	1	0.982 **	0.916 **	0.205 *	0.960 **	0.918 **
Severely Sick	−0.980 **	0.988 **	0.982 **	1	0.932 **	0.206 *	0.965 **	0.897 **
Daily Deaths	−0.951 **	0.922 **	0.916 **	0.932 **	1	0.293 **	0.939 **	0.887 **
Infection Rate (R)	−0.319 **	0.234 **	0.205 *	0.206 *	0.293 **	1	0.380 **	0.325 **
Percentage of Positive Tests	−0.990 **	0.967 **	0.960 **	0.965 **	0.939 **	0.380 **	1	0.922 **
Number of Positive Tests	−0.928 **	0.909 **	0.918 **	0.897 **	0.887 **	0.325 **	0.922 **	1

* *p* < 0.05; ** *p* < 0.01.

**Table 7:** Correlation between dose 3 and the sickness factors from 1 August 2021 to 19 October 2021.

	Dose 3 Aggregate Inoculation	Mildly Sick People	Moderately Sick People	Severely Sick People	Daily Deaths	Infection Rate (R)	Percentage of Positive Tests	Number of Positive Tests
Dose 3 Aggregate Inoculation	1	−0.813 **	−0.803 **	−0.657 **	−0.765 **	−0.820 **	−0.843 **	−0.770 **
Mildly Sick	−0.813 **	1	0.977 **	0.919 **	0.860 **	0.488 **	0.944 **	0.874 **
Moderately Sick	−0.803 **	0.977 **	1	0.930 **	0.875 **	0.492 **	0.940 **	0.868 **
Severely Sick	−0.657 **	00.919 **	0.930 **	1	0.837 **	0.236 *	0.882 **	0.793 **
Daily Deaths	−0.765 **	0.860 **	0.875 **	0.837 **	1	0.501 **	0.875 **	0.781 **
Infection Rate (R)	−0.820 **	0.488 **	0.492 **	00.236 *	0.501 **	1	0.536 **	0.517 **
Percentage of Positive Tests	−0.843 **	0.944 **	0.940 **	0.882 **	0.875 **	0.536 **	1	0.918 **
Number of Positive Tests	−0.770 **	0.874 **	0.868 **	0.793 **	0.781 **	0.517 **	0.918 **	1

* *p* < 0.05; ** *p* < 0.01.

**Table 8:** Regression between the severely sick patients and doses 1, 2, and 3.

	Severely Sick	Severely Sick	Severely Sick
Dose 1 Aggregate Inoculation	−30.167 **		
Dose 2 Aggregate Inoculation		−24.720 **	
Dose 3 Aggregate Inoculation			−9.377 **
R-square Value	0.894	0.961	0.431
Adjusted R-square Value	0.893	0.961	0.424
F	1173.6	3129.5	59.2
Significance of F	<0.01	<0.01	<0.01

** *p* < 0.01.

**Table 9:** Regression between the moderately sick patients and doses 1, 2, and 3.

	Moderately Sick	Moderately Sick	Moderately Sick
Dose 1 Aggregate Inoculation	−7.633 **		
Dose 2 Aggregate Inoculation		−5.887 **	
Dose 3 Aggregate Inoculation			−3.536 **
R-square Value	0.900	0.937	0.645
Adjusted R-square Value	0.899	0.937	0.641
F	33.2	1894.2	141.9
Significance of F	<0.01	<0.01	<0.01

** *p* < 0.01.

**Table 10:** Regression between the mildly sick patients and doses 1, 2, and 3.

	Mildly Sick	Mildly Sick	Mildly Sick
Dose 1 Aggregate Inoculation	−16.584 **		
Dose 2 Aggregate Inoculation		−12.614 **	
Dose 3 Aggregate Inoculation			−8.842 **
R-square Value	0.918	0.959	0.660
Adjusted R-square Value	0.917	0.958	0.656
F	1553.7	2935.8	151.5
Significance of F	<0.01	<0.01	<0.01

** *p* < 0.01.

**Table 11:** Regression between the daily deaths and doses 1, 2, and 3.

	Daily Deaths	Daily Deaths	Daily Deaths
Dose 1 Aggregate Inoculation	−1.386 **		
Dose 2 Aggregate Inoculation		−0.987 **	
Dose 3 Aggregate Inoculation			−0.561 **
R-square Value	0.909	0.905	0.585
Adjusted R-square Value	0.909	0.904	0.579
F	1392.2	1203.8	109.7
Significance of F	<0.01	<0.01	<0.01

** *p* < 0.01.

**Table 12:** Regression been the daily infection rates (R) and doses 1, 2, and 3.

	Infection Rate (R)	Infection Rate (R)	Infection Rate (R)
Dose 1 Aggregate Inoculation	−0.005 **		
Dose 2 Aggregate Inoculation		−0.003 **	
Dose 3 Aggregate Inoculation			−0.010 **
R-square Value	0.244	0.102	0.673
Adjusted R-square Value	0.238	0.095	0.669
F	44.8	14.4	160.4
Significance of F	<0.01	<0.01	<0.01

** *p* < 0.01.

**Table 13:** Regression between the daily percentage of people who tested positive and doses 1, 2, and 3.

	Percentage of Positive Tests	Percentage of Positive Tests	Percentage of Positive Tests
Dose 1 Aggregate Inoculation	−0.264 **		
Dose 2 Aggregate Inoculation		−0.213 **	
Dose 3 Aggregate Inoculation			−0.165 **
R-square Value	0.934	0.980	0.710
Adjusted R-square Value	0.934	0.980	0.706
F	1968.1	6142.5	191.1
Significance of F	<0.01	<0.01	<0.01

** *p* < 0.01.

**Table 14:** Regression between the daily number of people who tested positive and doses 1, 2, and 3.

	Number of Positive Tests	Number of Positive Tests	Number of Positive Tests
Dose 1 Aggregate Inoculation	−200.482 **		
Dose 2 Aggregate Inoculation		−146.884 **	
Dose 3 Aggregate Inoculation			−230.507 **
R-square Value	0.856	0.861	0.592
Adjusted R-square Value	0.855	0.860	0.587
F	826.4	787.2	113.2
Significance of F	<0.01	<0.01	<0.01

** *p* < 0.01.

## Data Availability

The data are available on the Israel Ministry of Health website (https://datadashboard.health.gov.il/COVID-19/general accessed on 24 January 2023).
